# Localisation of somatostatin and somatostatin receptors in benign and malignant ovarian tumours

**DOI:** 10.1038/sj.bjc.6600284

**Published:** 2002-07-15

**Authors:** G H Hall, L W Turnbull, I Richmond, L Helboe, S L Atkin

**Affiliations:** Department of Radiology, University of Hull, Centre for Magnetic Resonance Investigations, Hull Royal Infirmary, Anlaby Road, Hull, HU3 2JZ, UK; Department of Pathology, Hull Royal Infirmary, Anlaby Road, Hull, HU3 2JZ, UK; Neurobiology, H. Lundbeck A/S, Ottiliavej 7, DK-2500 Valby, Denmark; Department of Diabetes and Endocrinology, University of Hull, Michael White Diabetes Centre, Hull Royal Infirmary, Anlaby Road, Hull, HU3 2JZ, UK

**Keywords:** somatostatin, somatostatin receptors, ovarian cancer, immunohistochemistry, angiogenesis

## Abstract

Somatostatin has been identified as having anti-proliferative, anti-angiogenic and pro-apoptotic actions in many tumour systems, and these effects are mediated through a family of five transmembrane G-protein coupled SRIF receptors. Ovarian cancer is the commonest gynaecological malignancy in the UK and maintenance therapy is urgently required. Native somatostatin expression and its receptors sst_1,2,3 and 5_ were studied with immunohistochemistry in 63 malignant and 35 benign ovarian tumours of various histological types. Fifty-seven out of 63 (90%) of malignant and 26/35 (74%) benign tumours expressed somatostatin. Receptors sst_1,2,3 and 5_ were expressed variably in epithelial, vascular and stromal compartments for both benign and malignant tumours. Somatostatin was found to correlate significantly with stromal sst_1_ (*P*=0.008), epithelial sst_1_ (*P*<0.001), stromal sst_2_ (*P*=0.019), vascular sst_2_ (*P*=0.026), epithelial sst_3_ (*P*=0.026), stromal sst_5_ (*P*=0.013) and vascular sst_5_ (*P*=0.038). Increased expression of native somatostatin correlating with somatostatin receptors in malignant ovarian tumours raises the possibility that either synthetic somatostatin antagonists or receptor agonists may have therapeutic potential.

*British Journal of Cancer* (2002) **87**, 86–90. doi:10.1038/sj.bjc.6600284 www.bjcancer.com

© 2002 Cancer Research UK

## INTRODUCTION

Ovarian cancer is the commonest of the gynaecological malignancies in the western world with over 5000 new cases per annum in the UK (Office for National Statistics, 1998) and an overall 5-year survival of under 30%. Current therapy relies on debulking surgery with adjuvant chemotherapy, but relapse is common and development of an effective maintenance treatment is needed critically. Increased tumour vascular endothelial growth factor (VEGF) expression is associated with a poor prognosis (Paley *et al*, 1997) supporting the role of angiogenesis in the progression of this disease, *in vivo* neutralisation of VEGF with antisera has been shown to inhibit tumour growth and ascites (Olson *et al*, 1996). The regulatory tetradecapeptide somatostatin (SRIF), exhibits anti-proliferative (Buscail *et al*, 1994; Florio *et al*, 1996; Srikant, 1997), anti-angiogenic (Patel *et al*, 1994; Albini *et al*, 1999) and pro-apoptotic actions (Sharma *et al*, 1996). These effects are mediated through a family of five trans-membrane G-protein coupled somatostatin (SRIF) receptors, which have been cloned (Hoyer *et al*, 1995), activate multiple post-receptor signal transduction pathways (Patel, 1999). Synthetic SRIF analogues such as SMS-201-995 (Sandostatin, octreotide), RC-160 (Vapreotide, octastatin) and BIM-23014 (Lanreotide, somatuline) have been developed which have varying binding affinities for different receptor subtypes. They have been shown to potentiate the effects of tamoxifen in the inhibition of growth of mammary carcinomas in nude mice (Weckbecker *et al*, 1994) and to control growth of Kaposi's sarcoma by inhibition of angiogenesis (Albini *et al*, 1999). Recently in a cohort of 15 serous and two mucinous ovarian cystadenocarcinomas, 76% have been shown to demonstrate high affinity binding sites for the analogue RC-160 and RT–PCR has shown expression of mRNA for sst_1_ (65%), sst_2A_ (65%), sst_3_ (41%) and sst_5_ (24%) (Halmos *et al*, 2000). Therefore SRIF analogues may have a role as anti-angiogenic agents in the maintenance therapy of ovarian carcinoma. In order to explore the potential role of native SRIF in ovarian cancer, to further determine the localisation and expression of the SRIF receptors in a variety of ovarian neoplasms, we have examined the expression of both in a cohort of 63 malignant and 35 benign ovarian tumours, of various histological types, using immunohistochemistry.

## MATERIALS AND METHODS

### Experimental subjects

Permission was obtained from the local ethics committee to access material from the pathology archives at Hull and East Yorkshire NHS Hospitals Trust (Hull, UK). Representative paraffin blocks were taken from a cohort of 63 malignant and 35 benign ovarian tumours of mixed histological type. The mean age of patients studied was 57 years (range 30–84) with benign and 59 years (range 26–85) for malignant disease. According to Federation International of Gynaecology and Obstetrics (FIGO) classification for malignant ovarian disease 24 cases were stage I, seven were stage II and 32 were stage III. The histopathology of the tumours analysed with immunohistochemistry is summarised in Table 1

**Table 1:**
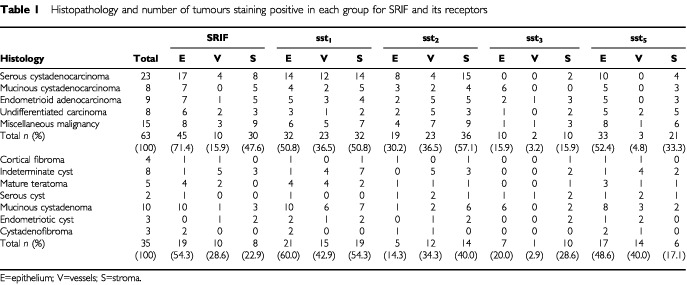
Histopathology and number of tumours staining positive in each group for SRIF and its receptors

. The wide mix of histological subtypes is representative of the breadth of ovarian tumours seen in clinical practice. The miscellaneous group of malignant tumours included two granulosa cell tumours, one carcinosarcoma, two clear cell adenocarcinomas, one malignant carcinoid tumour, one Leydig cell tumour and eight miscellaneous adenocarcinomas.

### Immunohistochemistry

Five-micrometer thick paraffin sections were dewaxed and antigen retrieval performed by microwaving at 600 W power in 10 mM citric acid for 20 min. Serial sections were stained with SRIF and SRIF receptor antibodies to facilitate comparisons between sections.

### Somatostatin

Tissue sections were pre-incubated in 10% non-immune goat serum (Dako Ltd, Ely, UK) for 20 min, then incubated with primary rabbit anti-SRIF antibody (AHP533, Serotec, UK) at a dilution of 1 : 40, overnight at 4°C. This antibody recognises both SRIF-14 and SRIF-28 variants. Sections were then washed with PBS, incubated with biotinylated goat anti-rabbit IgG (Dako Ltd, Ely, UK) at a dilution of 1 : 200 for 60 min, washed again with PBS and then HRP-StrepABC (PK-6100; Vector, Burlingame, CA, USA) was added for 45 min. Visualisation was achieved using DAB (Sigma-Aldrich Co Ltd, Poole, Dorset, UK) as an enzyme substrate, counterstained with haematoxylin, dehydrated and mounted.

### Somatostatin receptors

For SRIF receptor immunohistochemistry rabbit polyclonal antibodies to sst_1,2 and 5_ were produced and provided by Dr Helboe as previously described (Helboe *et al*, 1997). Rabbit polyclonal anti- sst_3_ antibody was obtained from Gramsch Laboratories (Schwabhausen, Germany). Sections were pre-incubated in 5% non-immune swine serum (Dako Ltd, Ely, UK) in PBS (pH 7.4) with 1% bovine serum albumin (BSA) and 0.3% Triton X-100 for 20 min at room temperature. Rabbit anti-SRIF receptor IgG was then added diluted in PBS plus 1% BSA and 0.3% Triton X-100, overnight at 4°C. Dilutions used were 1 : 8000 for sst_1_, 1 : 10 000 for sst_2_ 1 : 5000 for sst_3_ and 1 : 7000 for sst_5_. Sections were washed with PBS containing 0.25% BSA and 0.05% Tween-20, incubated with biotinylated swine anti-rabbit IgG (E0353, Dako Ltd, Ely, UK) at 1 : 500 dilution for 60 min at room temperature. Sections were then washed in PBS with 0.05% Tween-20, incubated with tyramide blocking buffer (supplied with the biotinylated tyramide kit; NEL700; NEN Life Science Products, Boston, MA, USA) for 20 min, then incubated with HRP-StrepABC (PK-6100; Vector, Burlingame, CA, USA) for 45 min. Sections were again washed with PBS containing 0.05% Tween-20, biotinylated tyramide was then added at 1 : 100 dilution for 10 min, sections were washed again in PBS containing 0.05% Tween-20, incubated with HRP-StrepABC for 45 min, washed again with PBS and signal visualised with DAB prior to counterstaining, dehydrating and mounting.

Positive control experiments included normal human anterior pituitary, which stained positively for all the SRIF receptors, normal human pancreas, which stained positively for native SRIF. Negative controls included adsorption studies as previously described (Helboe *et al*, 1997), which abolished positive staining. In addition omission of the primary antibody and incubation with 1% non-immune serum also abolished positive staining.

Staining was graded by intensity into negative, weak, moderate or strong and the pattern of staining described as either focal or uniform. The tissue compartments that stained were classified into stromal, epithelial or vascular and the intensity of staining was graded for each.

### Statistical analysis

Results were tabulated and data analysed using the SPSS statistical package (SPSS Professional Statistics, SPSS Inc., Illinois, USA). The χ^2^ square test was used for differences in staining between benign and malignant groups and a probability of *P*<0.05 was considered to be statistically significant. The Spearman Rank test was employed to determine correlations coefficients between native SRIF and its receptor expression.

## RESULTS

### SRIF expression

**Figure 1:**
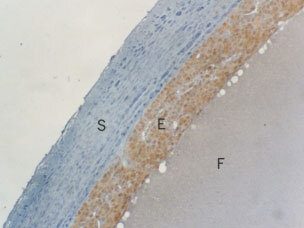
A serous cystadenocarcinoma demonstrating strong expression of SRIF by the malignant epithelium. S=Stroma, E=malignant epithelium, F=fluid in the cystic tumour (magnification ×200).

Fifty-seven out of 63 (90%) of malignant and 26 out of 35 (74%) of benign tumours expressed native somatostatin (SRIF). An example of a serous cystadenocarcinoma stained for SRIF is shown in Figure 1

. There was a trend toward more frequent expression in the epithelium of malignant tumours (71.4%) compared to benign (54.3%) although this failed to reach statistical significance (Table 1). There was significantly higher expression of SRIF in the vessels of benign tumours (28.6 *vs* 15.9%; *P*=0.044) but the stromal expression was significantly higher in the malignant tumours (47.6 *vs* 22.9%; *P*=0.005).

### SRIF receptor expression

**Figure 2:**
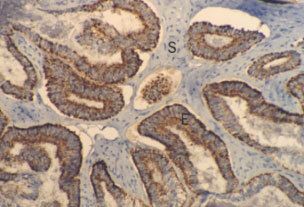
Ovarian endometrioid adenocarcinoma demonstrating uniform membrane bound epithelial staining for sst_5_. E=Epithelium; S=stroma (magnificatoin ×200).

Epithelial staining for the SRIF receptors was uniform where present. An endometrioid adenocarcinoma demonstrating epithelial expression of sst_5_ is shown in Figure 2

**Figure 3:**
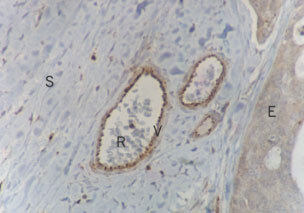
Endothelial sst_1_ expression in small tumour venules. V=Venule, R=red blood cells within vessel lumen, S=stroma, E=malignant epithelium (magnification ×400).

**Figure 4:**
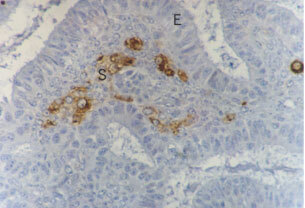
Ovarian adenocarcinoma demonstrating focal expression of sst_1_ on non-malignant stromal cells. There is both membrane bound and intracellular staining. E=Epithelium, S=stroma (magnification ×400).

. Vascular staining was largely found in the smooth muscle of the *tunica media* of arteries and veins and was fairly uniform, although some endothelial staining was also seen (Figure 3

). Stromal staining was more focal and patchy (Figure 4

).

Forty-eight out of 63 (76%) of malignant and 30 out of 35 (86%) benign tumours expressed sst_1_. There was significantly more frequent expression of sst_1_ in the epithelium (60%) of benign as compared with malignant (50.8%) tumours (*P*=0.034) although there were no differences between vascular and stromal staining (Table 1).

There was significantly more frequent expression of sst_2_ in malignant tumours in both the epithelium (30.2 *vs* 14.3%; *P*=0.044) and stroma (57.1 *vs* 40%; *P*=0.018) (Table 1). There was no difference in the vascular staining. It is of interest that five out of eight (62.5%) of undifferentiated carcinomas expressed vascular sst_2_ receptors. Overall 46 out of 63 (77%) of malignant tumours and 21 out of 35 (60%) of benign tumours expressed sst_2_ in at least one of the tissue compartments.

sst_3_ was the least expressed of the receptors studied, with overall only 18 out of 63 (29%) of malignant and 16 out of 35 (45%) of benign tumours expressing it. Both six out of eight (75%) mucinous cystadenocarcinomas and six out of 10 (60%) mucinous cystadenomas demonstrated epithelial sst_3_ (Table 1). Three tumours demonstrated vascular expression of sst_3_.

Forty-five out of 63 (71%) of malignant and 25 out of 35 (71%) of benign tumours demonstrated expression of sst_5_. There were no significant differences in epithelial or stromal expression between benign and malignant tumours, but benign tumours expressed significantly higher amounts of vascular sst_5_ (40 *vs* 4.8%; *P*<0.001) (Table 1).

### Correlation of SRIF with receptor expression

Native SRIF expression was found to correlate strongly with receptor expression in the same tissue compartments in both benign and malignant tumours, as follows: stromal sst_1_ (*P*=0.008), epithelial sst_1_ (*P*<0.001), stromal sst_2_ (*P*=0.019), vascular sst_2_ (*P*=0.026), epithelial sst_3_ (*P*=0.026), stromal sst_5_ (*P*=0.013) and vascular sst_5_ (*P*=0.038).

## DISCUSSION

The results form this large cohort of benign and malignant ovarian tumours show that both express high levels of native SRIF as well as the receptors sst_1,2,3 and 5_. We aimed specifically to determine whether malignant epithelium and blood vessels, as well as supporting stromal cells, expressed SRIF or its receptors. The presence of SRIF receptors on malignant epithelium is of great relevance to the potential use of SRIF analogues as chemotherapeutic agents, as is the presence of receptors on blood vessels for the potential use of SRIF analogues as anti-angiogenic therapy.

The expression of native SRIF by epithelial ovarian tumours is intriguing and its relationship with its receptor expression has not been well documented. SRIF occurs naturally in two forms, a 14 amino acid form (SRIF-14) and a 28 amino acid form (SRIF-28). Both are biologically active and bind to receptors. SRIF has previously been shown to be expressed by neuroendocrine ovarian carcinoid tumours (Sporrong *et al*, 1982). These tumours are exceptionally rare and demonstrate a different biological and clinical behaviour to the common epithelial ovarian tumours. SRIF mRNA production has been demonstrated in 14 out of 30 ovarian adenocarcinomas and two out of three borderline tumours (Reubi *et al*, 1993). In that study translation of mRNA was not confirmed by examining peptide expression and receptor autoradiography demonstrated SRIF receptors in only two of the 33 ovarian tumours. SRIF production has been demonstrated in the normal ovaries of many species, but in the human ovary it has only been demonstrated in follicular fluid to date (Holst *et al*, 1994). In this study we have examined the expression of both forms of SRIF in ovarian neoplasms, but have not examined expression in normal ovarian tissue.

As the actions of SRIF are inhibitory in most biological systems, it might have been expected that SRIF be expressed in benign lesions and this expression lost in malignancy. This was not the case, however, and raises further questions as to the role of SRIF in the pathophysiology of ovarian disease. Epithelial expression of SRIF was much greater than vascular or stromal, most of the SRIF staining was in the malignant cells themselves. The high levels of SRIF in ovarian malignancy may even suggest a stimulatory role in tumour growth through an autocrine positive feedback loop, perhaps involving up-regulation of receptors. This would not be unique, as SRIF-14 has been reported to stimulate tumour growth in the SHP-1 deficient pancreatic cell line MIA PaCa-2 whilst it inhibits growth in the SHP-1 positive PANC-1 cell line (Douziech *et al*, 1999). Further work is required to investigate the role of SHP-1 in ovarian cancer and to explore the actions of SRIF on the dynamics of tumour cells.

This study confirms that most ovarian tumours express SRIF receptors, but shows that there are differences in expression pattern between benign and malignant groups and between histological types. The malignant epithelium of ovarian tumours expresses high levels of sst_1, 2 and 5_ as well as SRIF itself. This suggests that SRIF may have a role in the progression of ovarian cancer. sst_3_ was only expressed in low amounts, as it is the receptor subtype thought to be most involved in stimulating apoptosis (Sharma *et al*, 1996), its low expression may be a significant factor in tumour progression. The strong correlations seen between SRIF and its receptors suggest that SRIF can cause up-regulation of its own receptors and be involved in auto-regulation of tumour growth. Of particular note is that vessels within even the most undifferentiated anaplastic tumours still express SRIF receptors and thus may be potential targets for therapy with the anti-angiogenic synthetic SRIF analogues.

An early report of SRIF receptor expression in ovarian tumours using *in vitro* receptor autoradiography found only 3/57 positive tumours (Reubi *et al*, 1991). A recent rapid communication of 15 serous and two mucinous cystadenocarcinomas, used radiolabelled RC-160 binding assays, specific for sst_2 and 5_, RT–PCR, to demonstrate expression of SRIF receptors in malignant ovarian tumours (Halmos *et al*, 2000). Whilst confirming that these two types of tumour expressed SRIF receptors, this methodology did not allow the anatomical localisation of receptor expression with respect to the malignant cells or surrounding normal stromal tissues. This is important to our understanding of the pathophysiology of the disease and how specific receptor targeting may act therapeutically.

SRIF analogues may exert their effects through both direct and indirect mechanisms (Pollak and Schally, 1998). Thus, even if tumour cells themselves do not express SRIF receptors, analogues may still inhibit tumour growth by indirect actions on other cells. One example of this is prevention of proliferation of an SRIF receptor-negative chondrosarcoma by the analogue SMS-201-995 via inhibition of growth hormone, insulin like growth factor-1 (IGF1) and insulin (Reubi, 1985). In Kaposi's sarcoma models, both *in vitro* and *in vivo,* SRIF has been shown to be a pure anti-angiogenic agent in its own right, inhibiting growth of SRIF receptor negative tumours (Albini *et al*, 1999). The stromal expression of SRIF and its receptors is important in many body systems, and is likely to be so in ovarian cancer too. The subtle interactions between malignant cells and their supporting stroma are poorly understood. Tumour associated macrophages have been reported in both benign and malignant ovarian tumours (Orre and Rogers, 1999), have been shown to have positive influence on tumour vascularisation. Some of the stromal cells expressing SRIF receptors (e.g. Figure 4) may be tumour-associated macrophages, it is possible that SRIF analogues might effect an action through this route. The demonstration of expression of SRIF receptors on stromal cells within ovarian tumours means that SRIF analogues could potentially alter tumour growth indirectly, by inhibiting stromal cell production of growth factors.

SRIF receptors have been described in both normal human blood vessels (Curtis *et al*, 2000) and veins surrounding human cancers (Reubi *et al*, 1994, 1996). IGF-1 stimulates growth of new blood vessels in experimental systems (Nakao-Hayashi *et al*, 1992) and potentially SRIF analogues may inhibit tumour growth indirectly by decreasing IGF-1 production. As SRIF receptors are expressed on peritumoral vessels they may act directly to inhibit angiogenesis or affect tumour biology by causing vasoconstriction and thus decreasing tumour blood flow (Reubi *et al*, 1996). The post-receptor signal transduction pathways in octreotide-induced inhibition of angiogenesis have been studied in the chick embryo system and have been shown to depend on G proteins, calcium and cyclic adenosine monophosphate (Patel *et al*, 1994). Our study has shown high-level expression of sst_1_ and sst_2_ in the vessels of both benign and malignant ovarian tumours, so there is potential for SRIF analogues to inhibit angiogenesis by both direct and indirect mechanisms. Vascular sst_5_ was expressed in 40% of benign and only 4.8% of malignant lesions, which may suggest that either the loss of sst_5_, which is postulated to have tumour suppressor actions, by benign vessels leads to the more rapid angiogenesis associated with malignancy, or that the increased production of SRIF by malignant lesions may lead to down-regulation of the vascular sst_5_ receptors.

Studies are already underway to look at the potential role of SRIF analogues in therapy of other solid tumours. SRIF analogues have been shown to be beneficial in a rat model where they potentiate the effects of tamoxifen (Weckbecker *et al*, 1994) and clinical trials in advanced breast cancer are underway (Bontenbal *et al*, 1998; O'Byrne *et al*, 1999). In ovarian cancer models the anti-angiogenic agents endostatin and angiostatin have been shown to act synergistically to inhibit tumour growth (Yokoyama *et al*, 2000). There is also evidence that the SRIF analogue RC-160 can inhibit growth of the ovarian cell line OV-1063 (Yano *et al*, 2000), but it cannot be extrapolated that this is true of all ovarian tumours *in vivo*. This gives hope that SRIF analogues may also prove efficacious by a combination of both the direct and indirect mechanisms. The cytotoxic SRIF analogue AN-238 has been shown to inhibit proliferation of SRIF receptor positive cells from the UCI-107 ovarian carcinoma cell line *in vitro* (Plonowski *et al*, 2001). Our study provides further rationale for exploring the potential therapeutic use of cytotoxic radionulide SRIF analogues in clinical trials of ovarian cancer.

We have shown the expression of SRIF and its receptors in both the epithelial and vascular compartments of benign and malignant epithelial ovarian tumours and have also noted significant stromal expression. It is likely that sst_1, 2 and 5_ will be more clinically important targets than sst_3_ for analogue mediated therapy. The role of SRIF and its receptors in the pathophysiology of ovarian disease requires further investigation as it may have either stimulatory or inhibitory actions.
